# 

**Published:** 1916-03-18

**Authors:** 


### PASSING EVENTS AND TOPICS.

### The King's Lancashire Military Convalescent Hospital.

As a tribute to the work which is being done at the Lancashire Military Convalescent Hospital, South Shore, Blackpool, the King has signified his pleasure that the hospital shall be styled in future the King's Lancashire Military Convalescent Hospital.

### Fire at Glasgow Hospital.

Damage estimated at £2,000 was done by an alarming fire which broke out in the laundry and wash-houses attached to the Victoria Infirmary, Glasgow. The fire spread to the electricity department, and thus cut off the light in the wards, which had to be illuminated by candles. No person was injured.

### Sketches of Serbia.

Two members of the Royal Free Hospital Serbian Unit, 1915—Mrs. Cora J. and Mr. Jan Gordon—opened on March 1 an exhibition of their sketches of Serbia, Montenegro, and Albania. The exhibition, which is being held at Walker's Galleries, 118 New Bond Street, W., will remain open until March 22.

### Provision for Soldiers on Discharge.

Sir Frederick Milner, Bart., who has been deputed by the King and Queen to visit all the hospitals in the country where the wounded are being nursed, has paid two visits to the Perth hospitals, and on both occasions has expressed great satisfaction with the arrangements. Sir Frederick delivers to the soldiers in hospital sympathetic Royal messages, and has drawn the attention of the men about to be discharged to the existence of the Soldiers and Sailors Help Society, a branch of which is established in different parts of the country, which is always ready to assist them during the period between the time of their discharge and the receipt of their pensions. Sir Frederick informed the wounded soldiers at Perth that he would be glad to help them if they had any difficulty in that matter, and stated that the men on their discharge were entitled to a bonus of £2.

### Windfall for King Edward's Fund.

King Edward's Hospital Fund benefits to the extent of something like £200,000 by the will of Isabella Countess of Wilton, who died on January 23 last, this being the estimated residue of the estate after various bequests have been deducted. In addition to this magnificent bequest, the Countess left £1,000 each to the Hospital for Epilepsy and Paralysis, Maida Vale, W.; the Fever Hospital, Islington; and the Taunton and Somerset Hospital, as well as varying sums to other charities. Indeed, with the exception of about £26,000, the whole of the estate—which is of the gross value of £290,000—is left to charity.

### The Cockerel and the Red Cross Fund.

One of the incidents of the recent auction of live stock at King's Lynn Cattle Market on behalf of the British Farmers' Red Cross Fund was the offering of a cockerel which had already brought £522 to the fund; this famous bird was sold and given back again several times, until the total amount for the day was brought up to over £85, when it was handed back to the fund. On the other hand, a dog, which apparently had points that failed to appeal to fanciers, was resold seventeen times for a grand total of 32s.

### Medical Appointments.

Lieut.-Col. W. H. Wilcox (R.A.M.C.), M.D., F.R.C.S. (senior out-patient physician to St. Mary's Hospital, has been appointed consulting physician to the Mesopotamia Expeditionary Force, with rank of colonel.

Dr. John Gilchrist, of Glasgow, has been appointed ophthalmic surgeon to the Victoria Eye Infirmary, Paisley, in succession to Dr. Cluckie, who is relinquishing the office owing to overstrain. Dr. Cluckie will, however, act as consulting ophthalmic surgeon.

Rates of Subscription: Twelve months, 6/6; Six months, 4/-; Three months, 2/-, post free to any address in the United Kingdom. Abroad, Twelve months, 8/8; Six months, 5/-; Three months, 2/6, post free.—Address The Subscription Dept., "The Hospital." The Hospital Building, 28/29 Southampton Street, Strand, London, W.C.

